# Study on the Strengthening Mechanism of Rare Earth Ce in Magnesium Alloys, Based on First-Principle Calculations and Electronegativity Theory

**DOI:** 10.3390/ma14216681

**Published:** 2021-11-05

**Authors:** Yanfei Chen, Zhengqiang Zhu, Jixue Zhou, Huasheng Lai

**Affiliations:** 1 School of Mechatronics Engineering, Nanchang University, Nanchang 330031, China; chenyf@sdas.org; 2 Advanced Materials Institute, Shandong Academy of Sciences, Jinan 250014, China; zhoujx@sdas.org; 3 Ganzhou Nonferrous Metallurgy Research Institute, Ganzhou 341400, China; songxc@sdas.org

**Keywords:** rare earth, magnesium alloy, cerium, strengthening mechanism, first principles

## Abstract

Since the commercial applications of rare earth magnesium alloys are increasing gradually, there are considerable advantages to developing lower cost and higher performance magnesium alloys with high abundance rare earth (RE) elements. However, the alloying order of a matrix magnesium alloy is completely changed with the addition of RE elements. Therefore, further study of the strengthening mechanism of Ce element in magnesium alloys is required. In this work, the thermodynamic stability of the possible second phases in a Mg-Al-Mn-Ce multicomponent magnesium alloy were analyzed, based on first-principle calculations, and the precipitation sequence of the key RE phases was deduced as a consequence. Combined with Scanning Electron Microscope (SEM), X-ray Diffractometer (XRD), Energy Dispersive Spectrometer (EDS), and other experimental methods, it was investigated whether the preferentially precipitated second phases were the nucleation core of primary α-Mg. The complex alloying problem and strengthening mechanism in a multi-elemental magnesium alloy system were simplified with the aid of electronegativity theory. The results showed that the preferentially precipitated Al_11_Ce_3_ and Al_10_Ce_2_Mn_7_ phases could not be the nucleation core of primary α-Mg, and the grain refinement mechanism was such that the second phases at the grain boundary prevented the growth of magnesium grains. Moreover, the tensile test results showed that the reinforced structure, in which the Al-Ce phase was mixed with Mg-Al phase, was beneficial for improving the mechanical properties of magnesium alloys, at both ambient temperature and high temperature.

## 1. Introduction

Lightweight materials have become an important development direction for the transportation and aviation industries [1]. As the lightest structural material, magnesium alloys have been increasingly used to replace some traditional materials in weight-critical applications, owing to their high specific strength/specific stiffness, recyclability, rich resources, and excellent process performance [2]. However, the poor heat resistance of magnesium alloy has limited its applications in certain fields [3]. According to the viewpoints in the literature [4,5], this was caused by the poor thermal stability of the strengthening phases in magnesium alloy (such as Mg_17_Al_12_, MgZn, Mg_3_Sb_2_, etc.), although they might perform well at ambient temperature. Pekguleryuz MO [6] suggested this was related to the activation of a slip system. As the slip system of magnesium alloy was only {0001}, <11$\overset{-}{2}$0> at ambient temperature, but the {10$\overset{-}{1}$0} {10$\overset{-}{2}$2} and {1010} sliding surfaces were all activated when the temperature reached 250 ℃, which made deformation much easier. Obviously, the activation of a slip system at high temperature is inevitable; therefore, how to synthesize a highly stable second phase during the smelting process, and the strengthening and toughening mechanisms of magnesium alloys, became the main directions of research [7,8].

With a unique 4f sub-layer electronic structure, RE elements are easy to form into intermetallic compounds with a strong interatomic bonding with Mg, Al, Mn, etc., which makes RE an ideal alloying element for magnesium alloys [7]. A large number of researchers have reduced the temperature sensitivity of magnesium alloys by adding rare earth elements such as Y, La, Ce, Pr, Nd, Gd, Dy, Sm, etc. [9], among which the most studied were Mg-Gd and Mg-Y series magnesium alloys [7], mainly because of their excellent aging and strengthening properties. By preparing Mg-Gd-x, Mg-Y-x ternary, or multiple alloy systems (x = Y [10,11,12], Sm [13,14], Nd [15], Zn [16,17,18], Ag [19,20,21], etc.), most researchers studied the structure and precipitation sequence of rare earth strengthening phases [22], explored the mechanical properties and fracture modes of samples with different heat treatments [23], and investigated the strengthening mechanism of rare earth elements in magnesium alloys [24,25].

What is certain is that, not only rare earth elements, but some conventional alloying elements (such as Si, Ca, Sr, etc.) also had the effect of improving the heat resistance of magnesium alloys. For example, adding Ca element to obtain Al_2_Ca phase [26,27], adding Si element to form Mg_2_Si phase [28,29], and adding Sr element to generate Al_4_Sr phase [30,31]. All these second phases had a relatively good thermal stability, but their precipitation at the grain boundary, on the other hand, significantly inhibited the continuous distribution of Mg_17_Al_12_, which improved the mechanical properties and the stability of the magnesium alloys during temperature cycling [26,27,28,29,30,31]. As a matter of fact, the strengthening mechanism of the second phases formed by conventional alloying elements and by rare earth elements was basically the same. However, the interatomic bonding force of the phases formed by rare earth elements was generally much higher than that of the conventional alloying elements [3], which meant that the thermal stability was higher and could be easily verified through first-principles calculations. Therefore, in order to obtain better heat resistance, the addition of more conventional alloying elements for modification was necessary, which made problems such as poor fluidity and hot cracking more likely [32]. Some literature reports [7,33] have pointed out that rare earth elements allowed bringing some challenging scientific and industrial magnesium alloys into reality, employing various complex design strategies, especially precipitation strengthening and grain refinement, which was an effective method for the preparation of special application magnesium alloys. Nevertheless, the addition of rare earth elements always increased the raw material cost; therefore, a potential alternative was to prepare application-oriented rare earth magnesium alloys by using high-abundance and low-cost rare earth elements, such as La, Ce, Y, etc. [23].

Cerium (Ce) is one of the RE elements with the highest contents [34], and whose atomic radius is close to that of Mg. The solid solubility of Ce in Mg was only 0.52% [35]. Therefore, the second phases formed by Ce were easily precipitated at the grain boundary, which hindered the slip of the grain boundary during deformation and effectively improved the mechanical properties and creep resistance of magnesium alloys. However, the addition of rare earth elements completely changed the alloying sequence of the matrix alloy, due to its unique physical, chemical, and electronegativity properties [36]. A large number of different types of second phases were formed as consequence [24]. Taking Mg-Ce and Al-Ce binary alloys as examples, the second phases found by experimental methods included MgCe [37], Mg_2_Ce [38], Mg_3_Ce [39], Mg_12_Ce [40], Mg_17_Ce_2_ [40], AlCe [41], Al_2_Ce [42], Al_3_Ce [43], Al_4_Ce [25], Al_11_Ce_3_ [44], etc. Although transmission electron microscopy (TEM), electron probe micro analyzer (EPMA), X-ray diffraction (XRD), differential scanning calorimetry (DCS), energy dispersive spectroscopy (EDS), and other techniques were used to analyze the structure and properties of these phases, the information on the formation sequence and thermodynamic stability of the phases was still limited. It should be noted that it took extensive work to verify the effect of these precipitations on magnesium alloys by experimental methods, one by one, not to mention that the β-phase precipitation sequence and the strengthening mechanism of Mg-Ce based ternary or multi-elements alloys were much more complicated.

Material calculation played an important role in predicting the properties and formation laws of the second phases in multi-element alloys [45]. CALPHAD and first-principles calculations are often used to predict the phase diagram and precipitation sequence of second phases in binary or ternary magnesium alloys [37,39]. For example, Hui Zhang [46] systematically studied the formation enthalpy, phonon spectrum, elastic constant, and thermodynamic properties of Mg-x binary alloys (x = As, Ba, Ca, Cd, Cu, Dy, Ga, Ge, La, Lu, Ni, Pb, Sb, Si, Sn, and Y) using first-principle and CALPHAD methods. In addition, the phase balances of Mg-Ca-Ce and Mg-Al-Ca ternary alloys were investigated as well, which helped to better understand the strengthening mechanism of rare earth elements. Yong Zhou [47], Z. W. Huang [48], and Zheng B [49] calculated the lattice parameters, cohesive energy, formation enthalpy, electronic structure, and elastic constant of Mg_17_Al_12_ [47,48,49], Mg_2_Si, Al_2_Y [48], Mg_2_Al_3_, Mg_2_Sn, MgZn_2_, Mg_2_Ni, Al_3_Ni [49], respectively, through CASTEP code. What is more, the evolution of the second phases in Al-Ce [50,51], Al-Nd [50], Al-Y, Al-Sc, Mg-Sc [52] binary alloy, Al-Mg-RE (RE = La, Ce, Pr, Nd, Sm) [51], and Al-Mn-Ce [53,54,55] ternary alloy was further analyzed using CALPHAD, which was difficult to derive and verify through experiment methods. However, it was unfortunate that most of the studies did not combine the structural evolution results from experiments with the results of the thermodynamic stability calculations, to deduce the microstructure regulation mechanism of the alloy. Hence, the research efficiency could be greatly improved and relatively accurate research results could be obtained by analyzing the strengthening and toughening mechanisms of magnesium alloys with the aid of material calculations.

In this work, a method for predicting the influence mechanism of the rare earth element Ce on the solidification process of magnesium alloys was systematically proposed, based on material calculations, which employed first-principle calculations, electronegativity theory, and mismatch theory. Furthermore, the results were verified through experimental measures. This was achieved in the following way: First, an in-depth investigation on the thermodynamic stability of the possible Al-Ce, Mg-Ce, and Mg-Al phases was performed with formation enthalpy, cohesive energy, and electronic structure results, and using first-principles calculations. As a result, the composition and precipitation sequences of the key rare earth phases were deduced. Then, whether the preferentially precipitated second phases were the nucleation core of the primary α-Mg was systematically studied, based on mismatch theory, and confirmed by the microstructure, composition, and other experimental methods. The grain modification mechanism of alloys was also revealed. Third, the complex alloy reactions in Mg-Al-Zn-Mn-Ce multicomponent magnesium alloy were associated with different temperature stages in a Al-Ce, Mg-Al, and Al-Ce-Mn phases diagram, and simplified with the aid of electronegativity theory. Accordingly, the strengthening mechanism of Ce element in magnesium alloys was deduced and verified through tensile tests, at both ambient temperature and high temperature.

## 2. Experimental and First-Principle Calculation Details

The main purpose of this work was to study the mechanism of influence of Ce element on the solidification process of magnesium alloy. Therefore, pure magnesium, aluminum, zinc blocks, and Mg-Mn master alloy, etc. were used to prepare a matrix magnesium alloy. The compositions of the prepared matrix alloy were analyzed with a Shimadzu PDA-7000 Optical Emission Spectrometer (OES) and the results are shown in Table 1. On the basis of the matrix magnesium alloy, rare earth magnesium alloys with a Ce content of 0.2 wt.%, 0.4 wt.%, 0.6 wt.%, 0.8 wt.%, 1 wt.%, and 3 wt.% were prepared by adding Mg-30 wt.% Ce master alloy. The melting temperature was set at 750 °C, and the protective atmosphere was a mixture of CO_2_ and SF_6_. After stirring continuously for 2 min, the Mg-Ce master alloy was added for 10 min, then slagging, standing, and casting in a metal mold preheated to 200 ℃ were carried out to obtain an ingot with a diameter of 100 mm.

The test samples were sampled from the same position of different ingots using a liner cutting machine. The microstructure of the metallographic samples was observed with a Nikon LV150NA optical microscope (OM, Nikon Instruments Inc., Melville, NY, USA) and ZEISS EVO MA 10 Scanning Electron Microscope (SEM, Carl Zeiss AG, Jena, Germany) after inlaying, grinding, polishing, and etching. The compositions were analyzed with the aid of an Oxford X-Max Energy Dispersive Spectrometer (EDS, Oxford Instruments plc, Oxford, UK) and Panalytical XPert Powder X-ray Diffractometer (XRD, PANalytical B.V, Almelo, The Netherlands), and the scan speed of XRD was set at 2 °C/min. For tensile tests at ambient temperature and high temperature, a Sansi CMT-5305GL type universal testing machine (Shenzhen Suns Technology Stock Co., Ltd., Shenzhen, China) was used, and the stretching rate was 2 mm/min. Micro hardness tests were performed with a HuaYing SHYCHVT-50Z type micro hardness tester (Laizhou Huayin Testing Instrument Co., Ltd., Laizhou, China) with a 1-Kg force load applied for 10 s, and the results were the average of 5 test points.

First-principle calculations in this work were performed using the Cambridge Sequential Total Energy Package (CASTEP) code [56], which was an implementation of the pseudo-potential plane-wave method, based on density functional theory (DFT). Pseudoatomic calculations were performed for Mg 3s^2^, Al 3s^2^3p^1^, and Ce 4f^1^5d^1^6s^2^ [50]. Obviously, energy-level overlap occurred in Ce atoms, according to the electronic configuration, which meant that spin magnetic moments were produced by the semi-full electrons in the 4f and 5d orbitals. Spin polarization was added and LDA+U with a Hubbard U value of 6.0 eV was used in order to correct the calculation results [50,57,58]. The exchange correction functional was considered using generalized gradient approximation (GGA) with the Perdew-Burke-Eruzerhof (PBE) functional. Vanderbilt-type ultrasoft pseudo potentials were employed to describe the Coulomb interactions between the valence electrons and ionic core. The energy calculations in the first irreducible Brillouin zone were conducted with a 12 × 12 × 8 k-point mesh, using a Monkhorst–Pack scheme. The cut-off energy value for the plane-wave basis set was selected as 380.0 eV. Structural optimization was carried out using Broyden–Flecher–Goldfarb–Shanno (BFGS) minimization, which was followed by calculations of total energy and electronic structure with a self-consistent field (SCF) tolerance of 1 × 10^−6^ eV/atom [49]. The tolerances of the geometry optimization were set as follows: the difference of the total energy within 5 × 10^−6^ eV/atom, maximum ionic Hellmann–Feynman force within 0.05 eV/Ǻ, maximum ionic displacement within 0.001 Ǻ, and the maximum tress within 0.01 GPa [57].

## 3. First-Principle Calculation of the Rare Earth Phases

**Figure 1:**
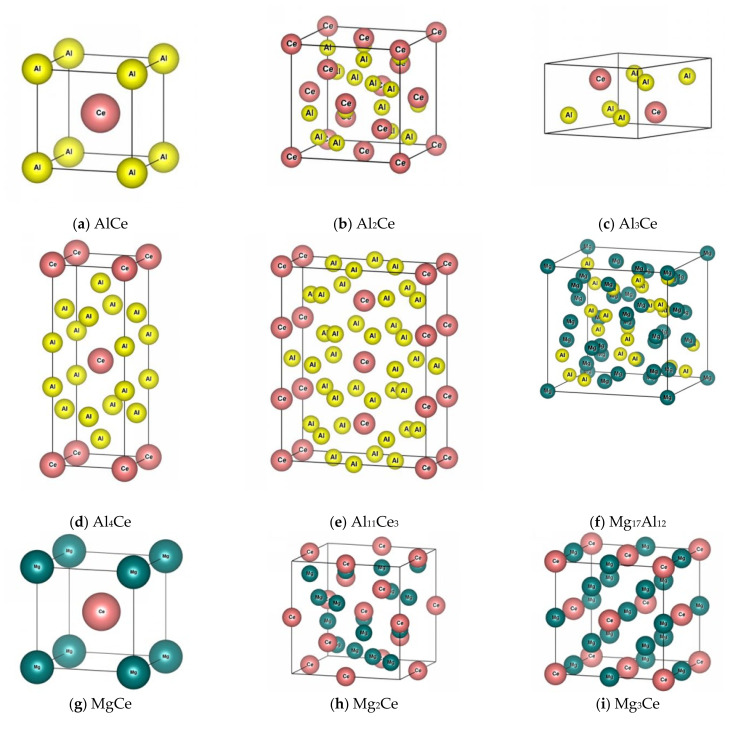

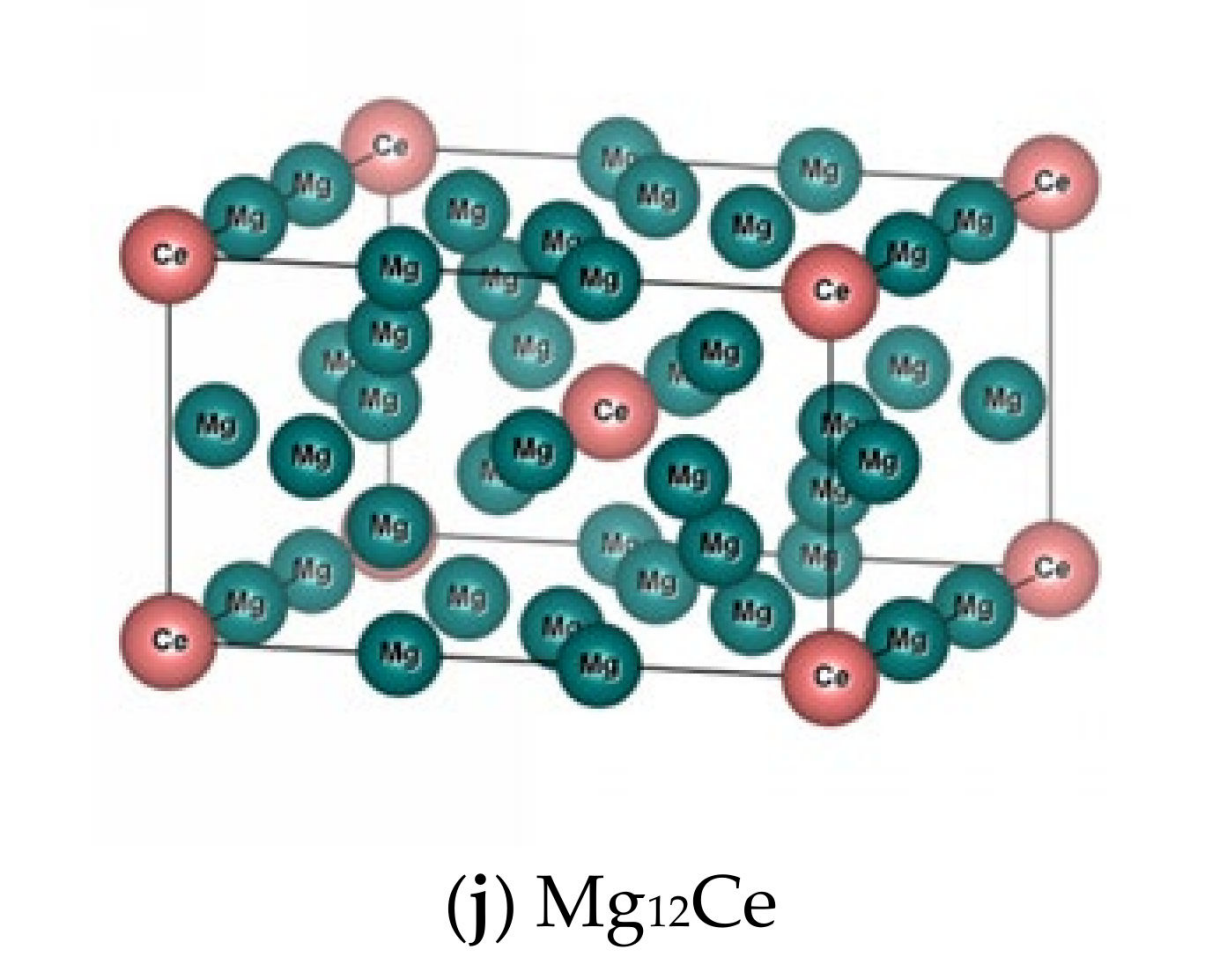
Crystal structure of the possible second phases in Mg-Al-Ce ternary alloy.

In a Mg-Al-Ce ternary alloy system, second phases can be one or more of the Mg-Al series, Mg-Ce series, or Al-Ce series. The crystal structures of the main second phases, according to the literature, are listed in Table 2; and the crystal models constructed with the structural information are shown in Figure 1. It was obvious that AlCe, Al_2_Ce, MgCe, Mg_2_Ce, Mg_3_Ce, and Mg_17_Al_12_ belonged to the cubic structure, which was a = b = c, α = β = γ = 90°; Al_3_Ce belonged to hexagonal structure, which was a = b ≠ c, α = β = 90°, γ = 120°; Al_4_Ce belonged to the square structure, with a = b ≠ c, α = β = γ = 90°; Al_11_Ce_3_ belonged to the orthogonal structure, with a ≠ b ≠ c, α = β = γ = 90°; and Mg_12_Ce belonged to a tetragonal structure, which was a = b ≠ c, α = β = γ = 90°. Among them, the spatial structures of AlCe and MgCe, and Al_2_Ce and Mg_2_Ce were exactly the same, except that the position of the Mg and Al atoms was exchanged.

To investigate the structural stability, the cohesive energy (E_Coh_) and formation enthalpy (△H) were calculated. The cohesive energy was defined as the total energy released by isolated atoms combined into a solid. It was used to characterize the strength of the bond between elements, which reflected the stability of the crystal structure [8]. While, the formation enthalpy referred to the energy absorbed in the process of forming a compound with a crystal structure from a pure element solid, which was used to reflect the difficulty of the formation of a second phase [34].

(1) $$\Delta H=\frac{1}{x+y}{(E}_{\mathrm{Total}}{-\mathrm{xE}}_{\mathrm{Solid}}^{A}{-\mathrm{yE}}_{\mathrm{Solid}}^{B})$$

(2) $$E_{\mathrm{Coh}}=\frac{1}{x+y}{(E}_{\mathrm{Total}}{-\mathrm{xE}}_{\mathrm{Atom}}^{A}{-\mathrm{yE}}_{\mathrm{Atom}}^{B})$$

The cohesive energy and formation enthalpy were calculated as Equations (1) and (2), respectively, where x and y were the numbers of A and B atoms in the unit cell, E_Total_ was the total energy of the unit cell, E^A^_Atom_ and E^B^_Atom_ were the energies of the atom in a free state, and E^A^_Solid_ and E^B^_Solid_ represented the energy of a pure element solid. A negative cohesive energy and formation enthalpy usually signified an exothermic process, and the lower the value, the stronger the stability. The results obtained through first-principle calculations are shown in Table 3 and Table 4, from which the stability and precipitation order of the second phases in the Mg-Al-Ce ternary alloy were preliminarily deduced [50,57,59,60].

Obviously, the E_Coh_ and △H of the Al-Ce series phases were both negative, indicating that the Al-Ce phase above could exist stably. Among them, Al_2_Ce had the largest △H, which was −0.488 eV; followed by Al_3_Ce, Al_11_Ce_3_, AlCe, and Al_4_Ce, with a △H of −0.407 eV, −0.365 eV, −0.333 eV, and −0.316 eV, respectively. The results showed a good agreement with the values from the research of Wen-Jiang Ding [57] and Michael C. Gao [50]. The formation enthalpy was arranged in the order Al_2_Ce > Al_11_Ce_3_ > Al_4_Ce in Wen-Jiang Ding’s research, and was expanded to Al_2_Ce > Al_3_Ce > Al_11_Ce_3_ > AlCe > Al_4_Ce by Michael C. Gao. Furthermore, the cohesive energy showed the same arrangement laws as the formation enthalpy [50,52,62].

**Figure 2:**
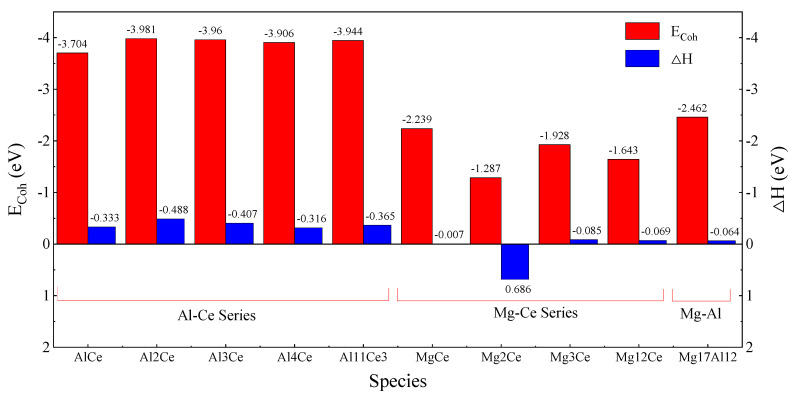
The contrast of E_Coh_ and △H among Al-Ce, Mg-Ce, and Mg-Al phases.

In contrast, the absolute values of E_Coh_ and the △H of Mg-Ce phases were significantly lower than that of the Al-Ce phases, indicating that the alloying ability and interatomic bonding of Mg and Ce elements were much lower, which was consistent with electronegativity theory. The △H of the Mg-Ce series phases decreased in the order Mg_3_Ce, Mg_12_Ce, and MgCe, with the values −0.085 eV, −0.069 eV, and −0.07 eV, respectively. While the △H of Mg_2_Ce was positive, which meant a difficulty in formation. In addition, the calculations showed that E_Coh_ and △H of the typical Mg_17_Al_12_ phase were negative, and the values were between those of the Al-Ce and Mg-Ce phases, indicating that the alloying ability between Mg and Al was lower than Al with Ce, but higher than Mg with Ce. A comparison of E_Coh_ and △H is shown in Figure 2.

The alloying reaction sequence of the Mg-Al-Ce ternary alloy system was deduced by comparing the E_Coh_ and △H results of the Mg-Ce series, Al-Ce series, and Mg-Al series intermediate phases. Since Ce had the strongest alloying ability, Ce was preferentially alloying with Al or other elements with a higher electronegativity difference (if any) to form a rare earth phase, until a certain element was exhausted. While, the remaining alloying elements reacted again, according to their alloying ability, until solidification. Combined with the addition of a small amount of Al (about 3 wt.%) and Ce (<1 wt.%) in this work, it could be inferred that the main precipitations of the Mg-Al-Ce ternary alloy were Al-Ce phase and Mg-Al phase. In addition, Ce element was added in a form of Mg-Ce master alloy at 750 ℃, which obviously did not meet the formation conditions of Al_2_Ce and Al_3_Ce (analyzed detailed in Section 4.3). Moreover, the micro equilibrium system was rich in Al; hence, the main Al-Ce intermediate phase was Al_11_Ce_3_, with a formation pathway of eutectic reaction at 641 ℃ [63].

## 4. Results and Discussion

### 4.1. Effect of Ce on the Microstructure and Compositions of Magnesium Alloys

**Figure 3:**
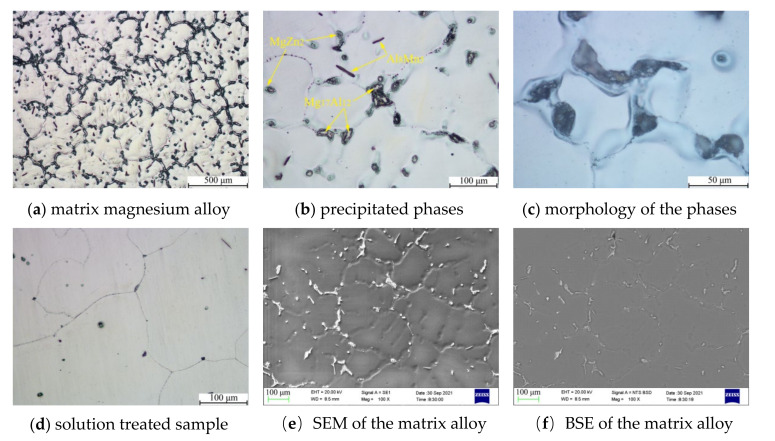
The microstructure morphology of Mg-Al-Zn-Mn-Si matrix alloy.

Metallographic diagrams of the matrix magnesium alloy with Al, Zn, Mn, and other elements added are shown in Figure 3. It was clear that the grains of the matrix magnesium alloy were equiaxed, with a size of about 600 μm. The second phases mainly precipitated continuously at the grain boundary, and a small fraction precipitated in the grain with a granular shape, as shown in Figure 3a–c. When the matrix alloy samples were solution treated at 200 ℃ for 20 h, the precipitated phases dissolved completely, as shown in Figure 3d. Rare earth magnesium alloys with Ce contents of 0.2 wt.%, 0.4 wt.%, 0.6 wt.%, 0.8 wt.%, 1 wt.%, and 3 wt.% (mass fraction) were prepared with this composition as a matrix in this work.

**Figure 4:**
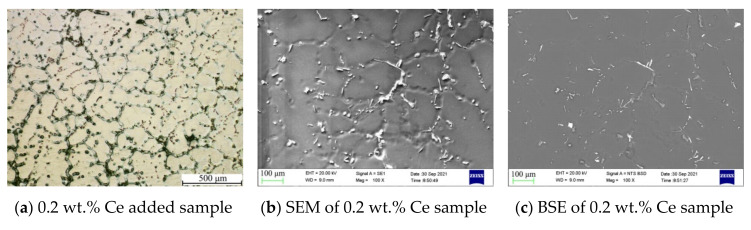

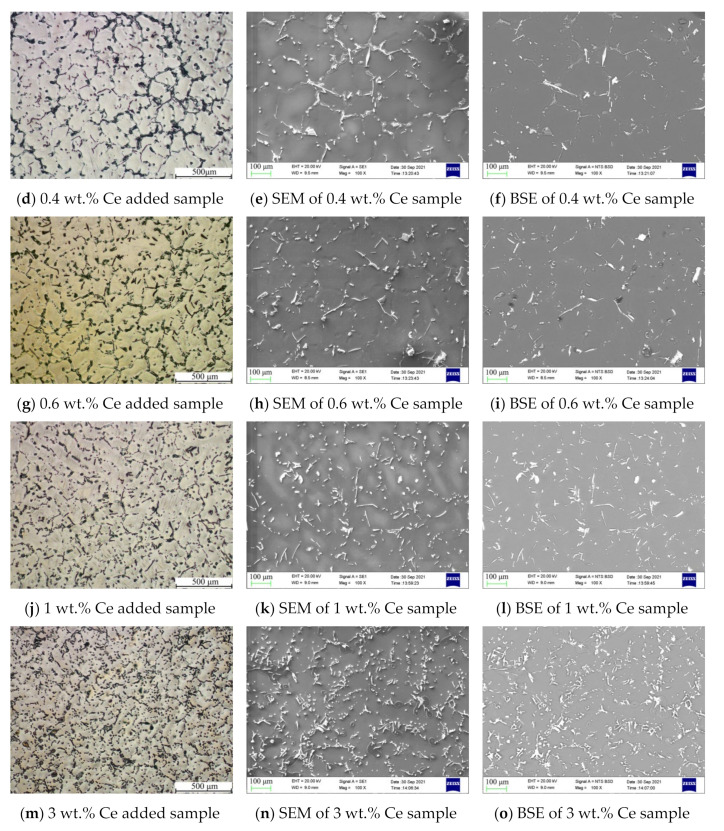
Metallographic and precipitated phase diagram of samples with different Ce contents.

The metallographic and precipitated phase morphology of magnesium alloys with 0.2–3 wt.% Ce added are shown in Figure 4. The average grain size decreased slightly to about 300–400 μm, while the number of small-sized grains increased after adding 0.2–0.4 wt.% Ce element. However, when the addition of Ce was more than 0.6 wt.%, the grains grew gradually, as shown in Figure 4g,j,m. What was noteworthy was that the precipitate structure changed significantly after the addition of Ce element, with a small number of needle-like precipitates appearing in the grain and grain boundary and mixed with the conventional granular precipitates. In addition, the number of needle-like precipitates rose gradually when the Ce content increased, as shown in Figure 4b,e,h.

**Figure 5:**
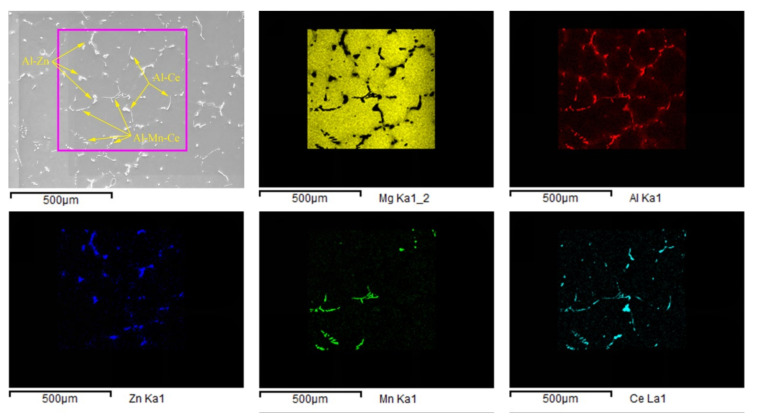
EDS surface scan results of the magnesium alloy with 0.2 wt.% Ce.

The back scattered electron (BSE) in Figure 4c,f,i,l and the EDS mapping observation in Figure 5 found several kinds of precipitates with obvious differences in shape and color contrast. It was clear that the Mn element appeared together with Al and Ce with a rod-like morphology, which was probably Al-Mn-Ce phase. The Zn element was accompanied by Al in the form of a granule shape (Al-Zn phase). While, needle-like phase with a length of about 50 μm was formed with Al and Ce elements (Al-Ce phase).

**Figure 6:**
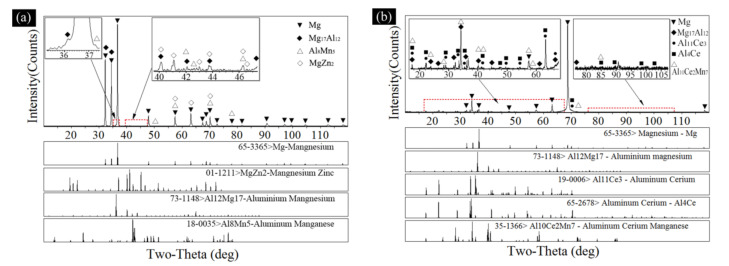
Composition of the alloys (**a**) matrix magnesium alloy (**b**) magnesium alloy with 0.8 wt.% Ce.

XRD was used to further analyze the compositions of the matrix alloy and samples with Ce element, and the results are shown in Figure 6. Clearly, the second phases in the matrix magnesium alloy were Mg_17_Al_12_, MgZn_2_, and trace Al_8_Mn_5_ (Figure 6a), which meant that the alloying reaction of the matrix alloy was mainly dominated by Al and Mg, and supplemented by the reaction of trace elements such as Zn, Mn, and Si.

The addition of Ce significantly changed the alloying sequence of the matrix magnesium alloy. Figure 6b shows the phase composition of the sample with 0.8 wt.% Ce. Combined with the EDS analysis in Figure 5, it can be seen that the Mg-Al phase in the EDS was actually Mg_17_Al_12_, the Al-Mn-Ce phase was Al_10_Ce_2_Mn_7_, and the Al-Ce phase was Al_11_Ce_3_ or Al_4_Ce phase. However, some trace phases were beyond the sensitivity of the XRD machine (such as Al-Zn, Mg-Si phase), which was difficult to calibrate but could be found in the EDS. The research results of Gao M C [50] showed that the Al_11_Ce_3_ was obtained by forming an Al vacancy in Al_12_Ce_3_ (i.e., Al_4_Ce); therefore, Al_11_Ce_3_ and Al_4_Ce had a quite similar structure. Moreover, the designed differential thermal experiment (DTA) showed that Al_11_Ce_3_ was formed by the eutectic transformation of Al_4_Ce at 641 °C. The overall reaction equation was Liq↔Al_11_Ce_3_+Al, which included Al_4_Ce↔Al+Al_11_Ce_3_. Therefore, Al_4_Ce was the high temperature β-phase of Al_11_Ce_3_, and the residue of Al_4_Ce was related to the solidification process. The XRD tests results showed a good agreement with the first-principle calculation results; both Al_11_Ce_3_ and Al_4_Ce could precipitate in Mg-Al-Ce ternary alloy, but the thermal stability of Al_4_Ce was not as good as Al_11_Ce_3_.

**Figure 7:**
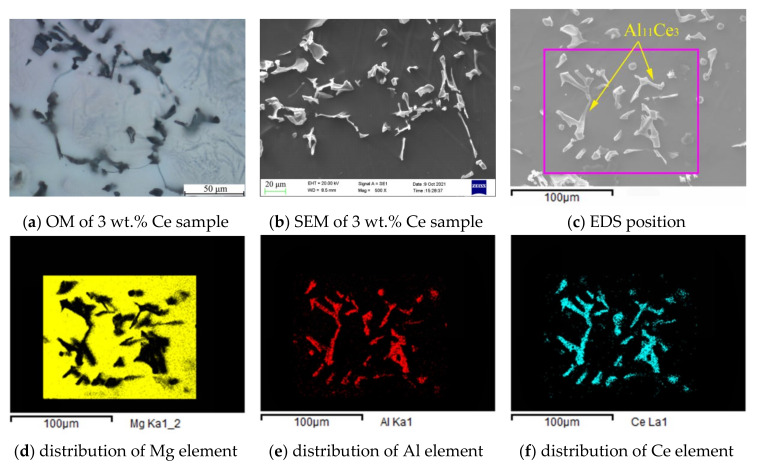

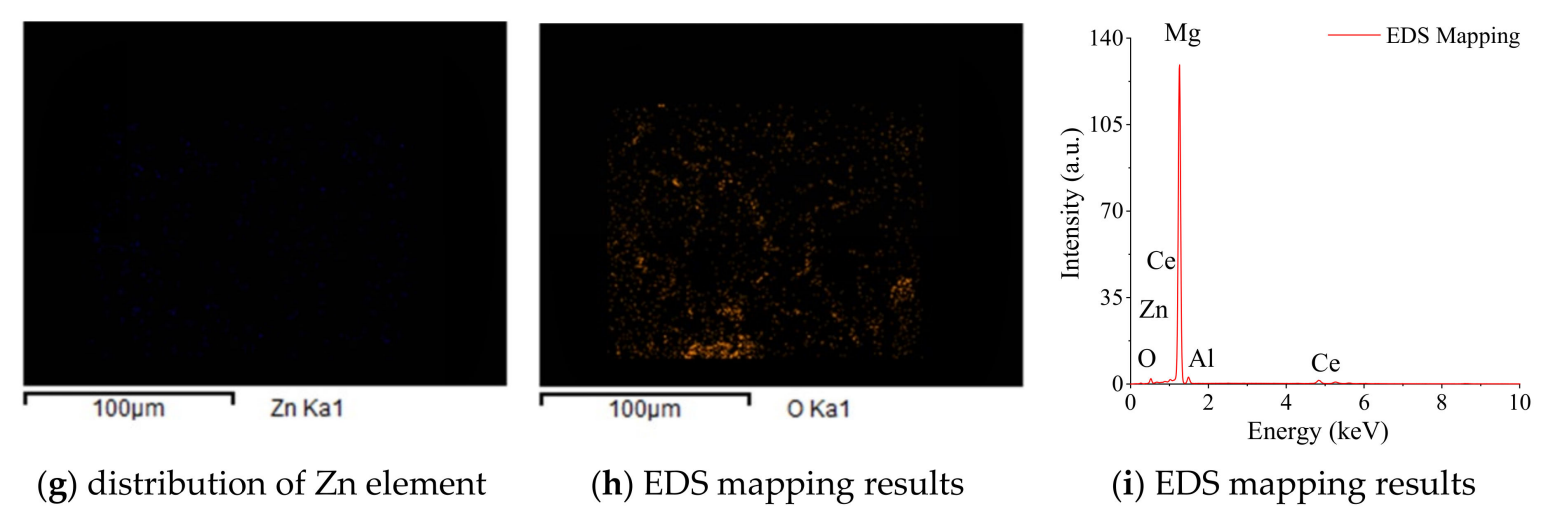
Morphology of the sample with 3 wt.% Ce added.

According to the electronegativity theory proposed by Linus Carl Pauling [64], the electronegativities of Mg, Al, Zn, Mn, and Ce were 1.293, 1.613, 1.59, 1.75, and 1.12, respectively. Obviously, the electronegativity difference between Ce and Mn was the largest, followed by Ce-Al, Ce-Zn, and Ce-Mg. Therefore, after the addition of Ce to the Mg-Al-Zn-Mn multi-elements system, Ce first alloyed with Mn, then with Al, Zn, and other elements, to form a rare earth phase, due to the electronegativity. Since the content of Mn in the matrix magnesium alloy was relatively low, only a small amount of Al_10_Ce_2_Mn_7_ phase was formed, preferentially with the depletion of Mn. An excess of Ce element alloying with Al and Al_11_Ce_3_ was formed as a result. Finally, the typical Mg_17_Al_12_ was formed by the remaining Al and Mg elements at about 436 °C, when the magnesium alloy had partially solidified. However, since the melting point of the Al_10_Ce_2_Mn_7_ phase was about 1150 °C [43,55] and its formation temperature was 700 °C, the magnesium alloy was still in liquid state [43] (see Section 4.3). Therefore, the formed Al_10_Ce_2_Mn_7_ phase floated or precipitated in liquid alloy, which could be removed by slag removal. The morphology and EDS results of the sample with 3 wt.% Ce are shown in Figure 7, it is clear that the precipitated phases became singular after slag removal; even though a large amount of rare earth elements were added at 750 °C, most of the precipitates were Al_11_Ce_3_ phases and only a small amount of other phases, such as Al-Zn, were detected. This analysis was verified by the EDS composition test results and was highly consistent with electronegativity theory and the first-principle calculations in the previous section.

Notably, the alloying reaction between Ce and the other alloying elements of the matrix reduced the number of nucleation cores, on the one hand, while the quantity of the second phases formed by other alloying elements was sharply decreased, on the other hand (such as Mg-Al, Al-Zn, Al -Mn, and Mg-Zn phases). However, the intermediate phases precipitated at the grain boundary could hinder the growth of crystal grains effectively, and also hindered the slip of the grain boundary when deformed. Therefore, after adding an excessive amount of Ce, the grain size of the magnesium alloy increased, and the mechanical properties of the alloy could be reduced, as shown in Figure 4d,g,j,m.

### 4.2. The Effect of Ce on the Mechanical Properties of Magnesium Alloys

**Figure 8:**
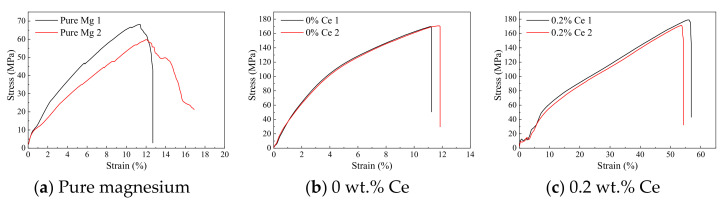

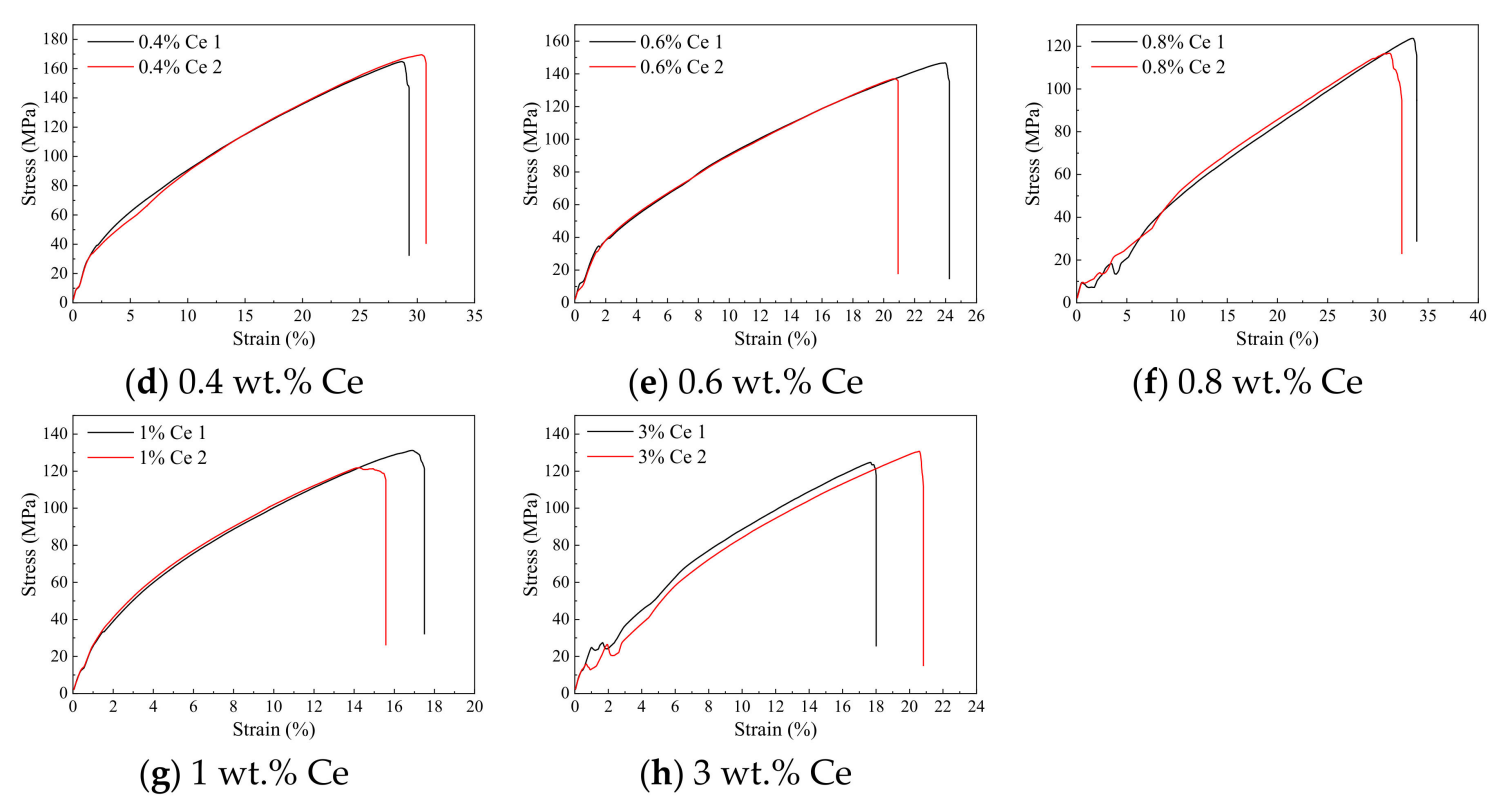
Tensile curves of the samples with different Ce contents at ambient temperature.

**Figure 9:**
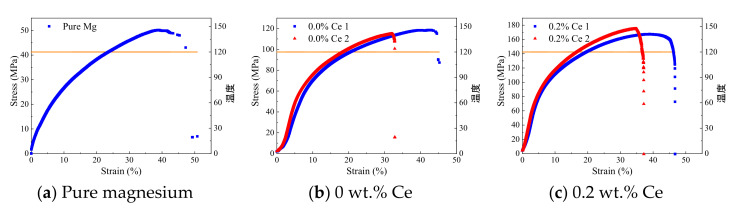

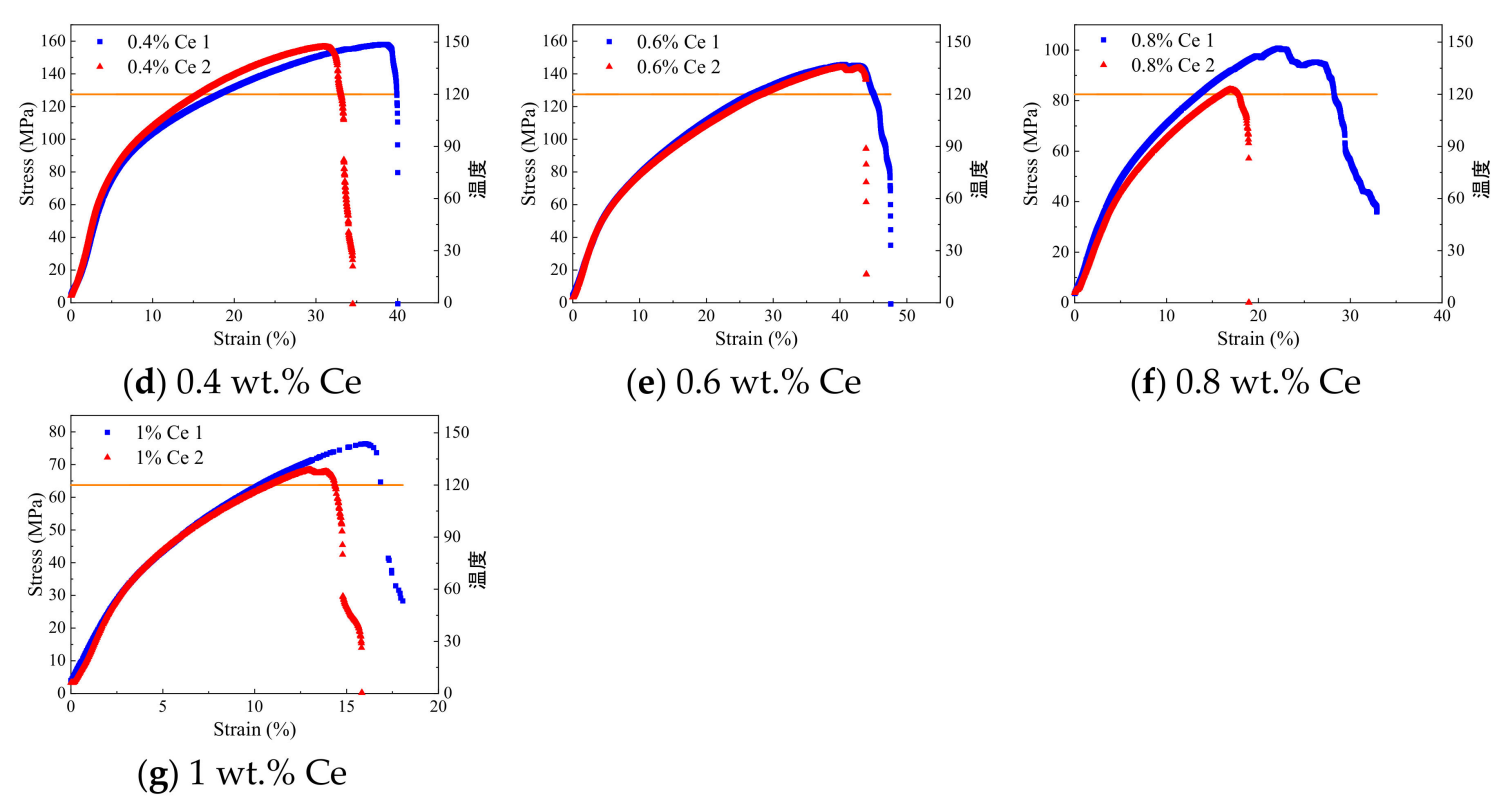
The tensile curves of the samples with different Ce content at high temperature. The Chinese on the right sides means Temperature.

Tensile samples were sampled from the same position of the ingots with different Ce contents, and stretched at both ambient temperature and 120 °C; the tensile curves are shown in Figure 8 and Figure 9. The tensile strength (σ_b_) of the as-cast pure magnesium was about 70 MPa, with an elongation (δ) of about 12% (obtained by the displacement of the chuck) at ambient temperature (Figure 8). As such, the mechanical properties of the pure magnesium were poor, and it would be difficult to meet the requirements of structural materials. However, the σ_b_ was improved to a level of 170 MPa after adding Al, Zn, Mn, and other alloying elements to the pure magnesium. However, the changes in δ were not that obvious, remaining at about 12%. When a further 0.2–0.4 wt.% Ce was added to the basis matrix magnesium alloy, the σ_b_ improved slightly to a 180 MPa level, but the δ increased remarkably to about 50%. Nevertheless, when the added amount of Ce exceeded 0.4 wt.%, the σ_b_ and δ both tended to decrease, which indicated that an appropriate amount of Ce was critical for the improvement of the plastic product strength.

The number of sliding systems of the magnesium alloy at ambient temperature was two, but the prismatic and conical sliding systems were activated at high temperature. As a result, the number of sliding systems was increased to five, which led to poor heat resistance. The tensile curves of the samples with different Ce contents at high temperature are shown in Figure 9, and the comparison data are listed in Table 5. It was obvious that the σ_b_ of the pure magnesium and matrix magnesium alloy decreased sharply, with a drop of about 40% (Figure 9a,b). However, both of them had a significant improvement in elongation, from 12% to 35%, which indicated the activation of the sliding system. Moreover, the above results meant that conventional elements such as Al, Zn, and Mn, etc. could not improve the high temperature performance of magnesium alloys. As a comparison, the samples with an appropriate amount of Ce were less sensitive to temperature. The σ_b_ of the sample with 0.2 wt.% Ce added at 120 °C was 10 MPa lower compared with that at ambient temperature, and the reduction was lower than 5%, which was maintained at a 170 MPa level (Figure 9c). Moreover, the elongation of the sample varied little and remained at about 40–50%, as at ambient temperature. The sample with 0.4 wt.% Ce added showed the same results, with a σ_b_ at 160 MPa level and a δ of about 35% (Figure 9d), which was 170 Mpa and 30%, respectively, at ambient temperature. The other samples with Ce added exhibited the same improvement in heat resistance. However, with the further increase in the addition of Ce, the tensile strength of the rare earth magnesium alloys at high temperature showed the same decreasing trends as at ambient temperature, which means that the mechanical properties were closely related to the mixed state of the rare earth phase with conventional phases.

**Figure 10:**
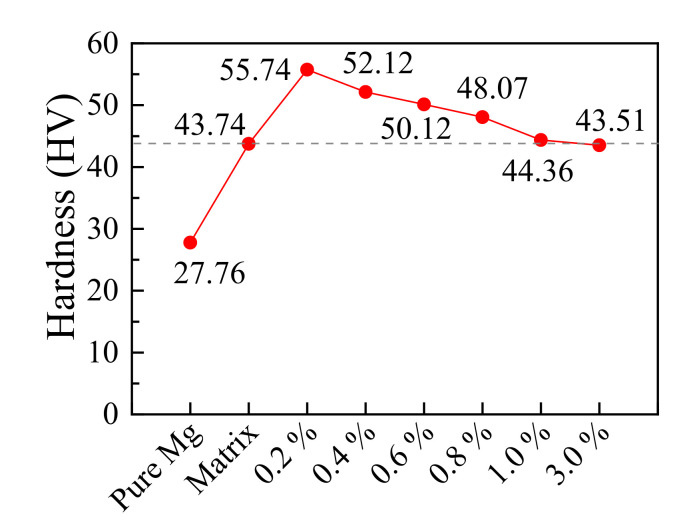
Hardness curve of the samples with different Ce contents.

Consistent with the tensile tests, the hardness of the alloy was also closely related to the precipitation state of the second phases [65,66]. Figure 10 shows the micro hardness curves of the samples with different Ce contents. It can be seen that the hardness of the samples with an appropriate amount of Ce element was much higher than that of the matrix alloy. Among them, the hardness reached a highest value of 55.74 HV when the Ce content was 0.2 wt.%, and where the precipitations were in a state in which a small number of rare earth phases were mixed with the conventional phases. However, as the content of Ce element increased, large numbers of conventional elements, such as Mn, alloyed with Ce and were precipitated [67], which resulted in a sharp decrease in the number of conventional phases. In addition, the hardness of the alloy gradually decreased as a result. When more than 3 wt.% Ce was added, the hardness of the sample started to become lower than that of the matrix magnesium alloy.

**Figure 11:**
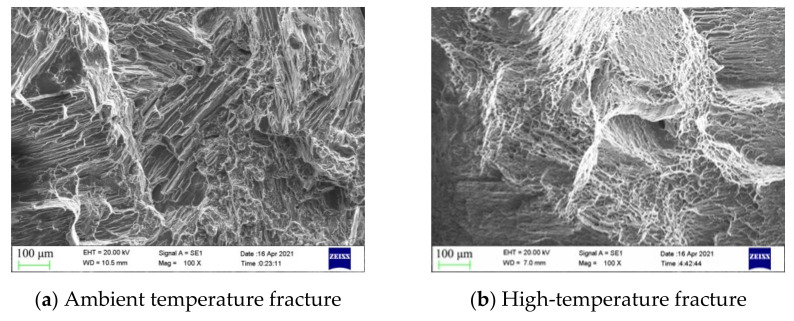
Fracture morphology of matrix magnesium alloy.

The ambient temperature fracture morphology of matrix magnesium alloy is shown in Figure 11a. A large number of fracture steps and tear ridges appeared in the fracture, which were typical brittle transcrystalline cleavage fractures. For comparison, high temperature fractures of the matrix magnesium alloy are shown in Figure 11b. Obvious small dimples and fracture steps appear at the fracture site, which prove that the high temperature activated the slip system, and that plasticity was exhibited to a certain degree. The fracture mode was a typical quasi-cleavage fracture, which is highly consistent with the high-temperature tensile results.

**Figure 12:**
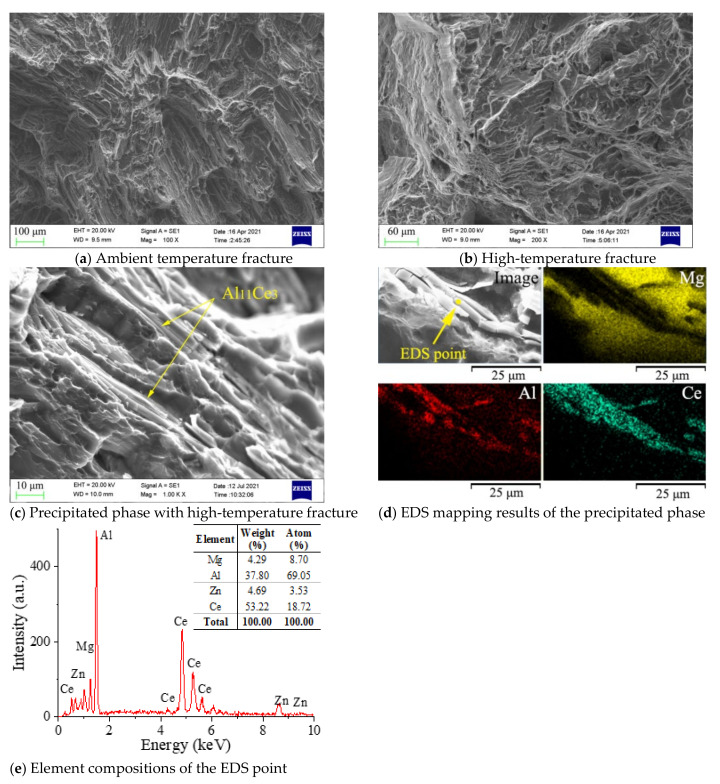
Fracture morphology of the sample with 0.2 wt.% Ce.

The fracture mode of magnesium alloys was not changed with the addition of Ce, as the fracture morphology of the sample with 0.2 wt.% Ce shows in Figure 12a,b. It was clear that the ambient temperature fractures of the Ce-containing sample had obvious tear ridges and fracture platforms, as in the sample without Ce, and the fracture mode was also brittle cleavage fractures. Tear ridges and a few dimples appeared in the high-temperature fracture, which proved it was a typical quasi-cleavage fracture. Whereas, the size of the dimples was larger than in the samples without Ce, indicating that the sample with Ce added had a better plasticity.

A magnified observation of the fracture found a needle-like second phase distributed at the bottom of the tear ridge, which was not fused with the matrix metal and penetrated the fracture with a broken shape. The EDS results showed that the phase was mainly composed of Al and Ce elements, with an atomic ratio of about 3:1 (Figure 12e), which suggests Al_11_Ce_3_ phase. The results above indicated that the high-temperature stable Al_11_Ce_3_ phase could hinder the activation of the Mg grain slip system during the high temperature deformation process, and the deformation resistance caused the fracture of Al_11_Ce_3_. However, the needle-shaped Al_11_Ce_3_ was prone to stress concentration and cracks, since the Al_11_Ce_3_ phase was not fused with the matrix alloy. Therefore, tensile fractures appeared at the interface of the Al_11_Ce_3_ and the matrix metal, which suggested that more Al_11_Ce_3_ was not always beneficial.

### 4.3. The Strengthening Mechanism of Ce in the Magnesium Alloy of Mg-Al Series

According to electronegativity theory [64], in the Mg-Al-Zn-Mn-Ce multi-elements alloying system in this work, the electronegativity difference between Ce with Mn, Al, Zn, and Mg decreased gradually. Therefore, the Ce preferentially alloyed with Mn to form Al_10_Ce_2_Mn_7_ phase. Owing to the low content of Mn in the matrix magnesium alloy, the excess Ce then alloyed with Al, Zn, Mg, and other elements sequentially to form the corresponding Al-Ce, Zn-Ce, and Mg-Ce phases, until the Ce was exhausted. However, the Al added to the matrix magnesium alloys was generally sufficient to completely consume all of the Ce element, and Al_11_Ce_3_ was formed as a result. Therefore, it was difficult to find Zn-Ce and Mg-Ce phases in the alloy, while the remaining Al alloyed with Mg to form Mg_17_Al_12_. This derivation is consistent with the component analysis results obtained previously in this study.

By using an edge–edge matching model [68], a set of crystal orientation relationship data was obtained by calculating the crystal plane spacing mismatch between the close-packed surfaces of α-Mg with Al_10_Ce_2_Mn_7_ and Al_11_Ce_3_ phases. Therefore, whether the preferentially precipitated Al_10_Ce_2_Mn_7_ and Al_11_Ce_3_ served as the nucleation core of α-Mg was investigated. Taking Al_11_Ce_3_ as an example, the three close-packed surfaces of α-Mg were {0002}, {1011}, and {1010}, and the three close-packed surfaces of Al_11_Ce_3_ were {011}, {002}, and {101}, respectively. According to the mismatch in Equation (3), the calculated crystal surfaces mismatch (f_d_) between α-Mg and Al_11_Ce_3_ is shown in Table 6.

(3) $$\begin{array}{l} f_{d}\;=\;\left|\frac{d_{Mg}\;-\;d_{{Al}_{11}{\mathrm{Ce}}_{3}}}{d_{Mg}}\right|\;\times \;100\% \end{array}$$

The mismatch between α-Mg and Al_10_Ce_2_Mn_7_ was calculated using the same method. The mismatches of α-Mg with Al_11_Ce_3_ and Al_10_Ce_2_Mn_7_ were much greater than 25%, which meant that they could not act as the nucleation core for α-Mg. The grain refinement mechanism after the addition of Ce element was primarily that the precipitated phases distributed along the grain boundaries hindered the growth of the grains. Therefore, the effect of Ce on the grain refinement of magnesium alloys was not satisfactory. On the country, after adding an excessive amount of Ce element, the number of other intermediate phases and particles that should have been precipitated in the matrix magnesium alloy was sharply reduced, due to the preferential alloying of Ce with Mn, Al, Zn, etc., and these phases could act as nucleation cores or hinder the growth of grains at the grain boundary. Therefore, the grain size increased instead. This was verified by the microstructure morphology in Figure 4 and discussed in Section 4.1.

**Figure 13:**
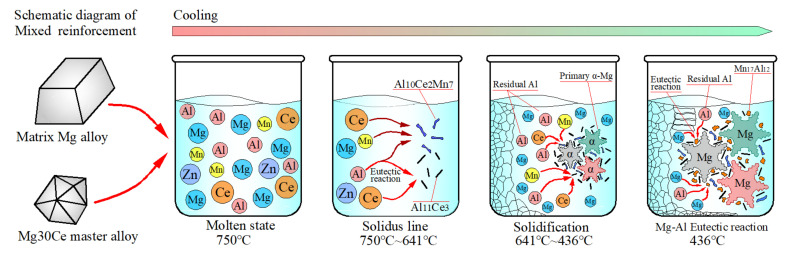
The strengthening mechanism of Ce element in the solidification process of Mg-Al alloys.

Based on the above theoretical analysis results, and combined with the Al-Ce and Mg-Al binary phase diagrams, and Al-Mn-Ce ternary phase diagrams, the strengthening mechanism of Ce element in the solidification process of Mg-Al alloys was deduced, which was divided into three stages, according to the temperature range, as shown in Figure 13.

(1) Between 750 °C and 641 °C: According to the research results of Qiang Yang [55], Al_10_Ce_2_Mn_7_ was transformed from Al_8_CeMn_4_. Gil Coury [53] confirmed that the two Al atoms in Al_10_CeMn_2_ were replaced by Mn atoms and converted into Al_8_CeMn_4_, which were observed using the TEM method, with both of them having similar properties and the melting point of Al_8_CeMn_4_ being 1191 °C. F. G. Coury [69] found that Al_8_CeMn_4_ was more likely to form at the Al-rich corner in Al-Mn-Ce ternary alloys, which was similar to the situation in this paper. The phase diagram calculations of Y. Yang [70] showed that Al_10_CeMn_2_ was transformed at 738 °C and 700 °C. Therefore, it could be inferred that the formation temperature of Al_8_CeMn_4_ (which could be converted into Al_10_Ce_2_Mn_7_) was also around 700 °C. Combined with electronegativity theory, it was suggested that the Al_10_Ce_2_Mn_7_ phase was preferentially formed from Al, Ce, and Mn elements, and solidified and dispersed at about 700 °C, when the matrix magnesium alloy was still in a liquid state. However, more specific formation temperatures and forming theories have rarely been reported [38,41], and need further study.
(2) Between 641 °C and 436 °C: The liquidus of magnesium alloy was about 650 °C, and the Al_11_Ce_3_ was precipitated through a eutectic reaction Liq↔Al_11_Ce_3_+α-Al at 641 °C [42]. However, the preferentially precipitated Al_10_Ce_2_Mn_7_ and Al_11_Ce_3_ phases could not act as the nucleation particles of α-Mg, according to the mismatch calculations results. Therefore, the precipitated Al_11_Ce_3_ and Al_10_Ce_2_Mn_7_ phases adhered to the α-Mg surface and were distributed along the grain boundary, which inhibited the growth of the primary α-Mg grains, thus achieving the effect of grain refinement. Whereas, the Ce content was less than 1 wt.%, the Al element in the matrix alloy was sufficient to fully alloy with Ce, and the residual Al then further alloyed with Mg, following the order in electronegativity theory.
(3) Between 436 °C and ambient temperature: the typical Mg_17_Al_12_ was generated at 436 °C through an eutectic reaction Liq↔Mg_17_Al_12_+α-Mg [47]. Since Al_10_Ce_2_Mn_7_ and Al_11_Ce_3_ had already been distributed along the grain boundary at this temperature, this blocked the Mg_17_Al_12_ from forming a network/continuous morphology. Therefore, the precipitated Mg_17_Al_12_ phase after the addition of Ce was mainly in the shape of islands or clumps at the grain boundary, and a mixed strengthening structure of rare earth phase and conventional phase was formed. As a matter of fact, the melting point of Mg_17_Al_12_ was only 467 °C, whereas the melting point of Al_10_Ce_2_Mn_7_ and Al_11_Ce_3_ was above 1000 °C [43,50,55]. In addition, the Ce-containing rare earth phase was not solid soluble in magnesium, and the heat resistance and high temperature strength of the magnesium alloy were significantly improved by adding an appropriate amount of Ce. However, with the increased addition of Ce, a large amount of conventional alloying elements (Mn, Al, Zn, etc.) were consumed. As a result, the strengthening phases in the magnesium alloy gradually changed from a mixed strengthening structure of rare earth and conventional precipitated phases to a single rare earth phase structure. However, the acicular Al_11_Ce_3_ phase was not fused with the magnesium matrix, which generated stress and formed microcracks. Therefore, excessive Ce element addition reduces the mechanical properties of an alloy.

In conclusion, the best way to improve the mechanical properties of Ce rare earth magnesium alloys is to adjust the proportion of Ce element added, so as to form a mixed strengthening structure of rare earth phase with conventional strengthening phase, which is of great significance for the design of magnesium alloys.

## 5. Conclusions

(1) The first-principle calculation results showed that the formation enthalpy and cohesive energy of the Al-Ce series phase were much higher than that of the Mg-Al and Mg-Ce series phases in a Mg-Al-Ce ternary alloy system, which meant that Ce would preferentially alloy with Al element to form Al-Ce phase. While Mg-Al phase and Mg-Ce phase would be formed in sequence when the Ce was completely consumed. The calculation results were highly consistent with electronegativity theory.
(2) The microstructure and composition analyses of the alloy found that the strengthening phase of magnesium alloy after adding Ce element was mainly needle-like Al_11_Ce_3_; rod-like Al_10_Ce_2_Mn_7_, which was distributed at the grain boundary and through the grains; and Mg_17_Al_12_ phase, which was broken into granular or island shapes at the grain boundary. In combination with mismatch theory, it was proven that the preferentially precipitated Al_11_Ce_3_ and Al_10_Ce_2_Mn_7_ phases could not act as the nucleation core of α-Mg, but instead were precipitated at the grain boundary, thereby blocking the continuous distribution of Mg_17_Al_12_. While, a reinforced structure was formed, with the rare earth phase mixed with conventional phase.
(3) The tensile results at ambient temperature and high temperature showed that a strengthened structure of rare earth phase mixed with conventional strengthening phase was beneficial for improving the comprehensive mechanical properties of magnesium alloys, by adding an appropriate amount of Ce element; with the appropriate Ce addition range being 0.2–0.4 wt %. After adding an excessive amount of Ce element, the strengthening phase of the alloy was mainly Al_11_Ce_3_. While, the acicular Al_11_Ce_3_, which did not fuse with the matrix, easily became the source of cracking, due to the stress concentration, and reduced the mechanical properties of the alloy.
(4) Combined with electronegativity theory, the mechanism of the microstructural evolution of a Mg-Al-Zn-Ce-Mn multi-elements alloy during solidification was simplified, with temperature as the dimension, and the strengthening mechanism of Ce element in magnesium alloys was deduced.

## Figures and Tables

**Table 1:** Composition of the matrix magnesium alloy (wt.%).

Elements	Al	Zn	Mn	Si	Fe	Cu	Mg
**wt.%**	3.156	1.098	0.385	0.167	0.029	0.014	Balance

**Table 2:** The possible second phases in Mg-Al-Ce ternary alloy.

**Species**	**AlCe**	**Al_2_Ce**	**Al_3_Ce**	**Al_4_Ce**	**Al_11_Ce_3_**
**Structure**	Pm$\overset{-}{3}$m	Fd$\overset{-}{3}$m	P63/mmc	I4/mmm	Immm
**Space No.**	221	227	194	139	71
**Species**	**MgCe**	**Mg_2_Ce**	**Mg_3_Ce**	**Mg_12_Ce**	Mg_17_Al_12_
**Structure**	Pm$\overset{-}{3}$m	Fd$\overset{-}{3}$m	Fm$\overset{-}{3}$m	I4/mmm	I$\overset{-}{4}$3m
**Space No.**	221	227	225	139	217

**Table 3:** The energy of atoms in a ground state and pure element solid.

Species	Mg Atom	Al Atom	Ce Atom	Mg Solid	Al Solid	Ce Solid
**E_Atom_/E_Solid_ (eV)**	−52.670	−972.494	−1058.339	−56.404	−973.949	−1061.349
**Cal.**	−52.738 ^a^	−972.226 ^a^	−1058.349 ^b^	−56.420 ^a^	−973.996 ^a^	−1064.147 ^b^

^a^ Ref. [49], ^b^ Ref. [61].

**Table 4:** E_Coh_ and △H of the possible second phases.

Species	AlCe	Al_2_Ce	Al_3_Ce	Al_4_Ce	Al_11_Ce_3_	MgCe	Mg_2_Ce	Mg_3_Ce	Mg_12_Ce	Mg_17_Al_12_
**△H (eV)**	−0.333	−0.488	−0.407	−0.316	−0.365	−0.007	0.686	−0.085	−0.069	−0.064
**Cal. (eV)**	−0.337 ^c^	−0.462 ^c^	−0.424 ^c^	−0.302 ^c^	−0.359 ^c^	−0.010 ^b^	1.311 ^b^	−0.080 ^b^	−0.061 ^e^	−0.053 ^d^
**E_Coh_ (eV)**	−3.704	−3.981	−3.960	−3.906	−3.944	−2.239	−1.287	−1.928	−1.643	−2.462
**Cal. (eV)**	-	-	-	-	-	−3.059 ^b^	−1.288 ^b^	−2.453 ^b^	-	−2.385 ^d^

^b^ Ref. [61], ^c^ Ref. [50], ^d^ Ref. [47], ^e^ Ref. [60].

**Table 5:** Comparison of UTS and elongation at ambient temperature and high temperature.

Species		Pure Mg	0 wt.% Ce	0.2wt.% Ce	0.4 wt.% Ce	0.6 wt.% Ce	0.8 wt.% Ce	1 wt.% Ce	3 wt.% Ce
Ambient Temp.	σ_b_ (MPa)	68.2	170.3	175.2	167.2	141.8	120.2	122.5	123.7
	δ (%)	11.8	11.5	54.9	29.6	22.4	32.3	16.2	19.3
High Temp.	σ_b_ (MPa)	50.1	116.6	171.0	157.2	144.7	110.7	72.3	-
	δ (%)	38.6	39.8	41.6	36.1	44.1	23.0	15.5	-

**Table 6:** The crystal surfaces mismatch between α-Mg and Al_11_Ce_3_.

*F_d_* (%)		*d_Mg_*		
		2.6050	2.4520	2.7782
***d_Al_11_Ce_3__***	7.9776	206.24	225.35	187.15
	5.0460	93.70	105.79	81.63
	4.0295	54.68	64.34	45.04

## Data Availability

Not applicable.
